# Inflammatory pseudotumor of the liver diagnosed as metastatic liver tumor in a patient with a gastrointestinal stromal tumor of the rectum: report of a case

**DOI:** 10.1186/1477-7819-12-140

**Published:** 2014-05-06

**Authors:** Yoichi Matsuo, Mikinori Sato, Takahiro Shibata, Mamoru Morimoto, Ken Tsuboi, Tomoya Shamoto, Takahisa Hirokawa, Takafumi Sato, Hiroki Takahashi, Hiromitsu Takeyama

**Affiliations:** 1 Department of Gastroenterological Surgery, Nagoya City University Graduate School of Medical Sciences, Kawasumi 1, Mizuho-cho, Mizuho-ku, Nagoya, 4678601, Japan

**Keywords:** Inflammatory pseudotumors of the liver, Malignant gastrointestinal stromal tumor, FDG-PET

## Abstract

**Background:**

Inflammatory pseudotumor (IPT) of the liver is a rare benign lesion. A case of IPT of the liver found in association with a malignant gastrointestinal stromal tumor (GIST) is reported.

**Case report:**

A 74-year-old man was admitted to our hospital for a liver tumor. He previously underwent rectal amputation for a malignant GIST. Computed tomography (CT) revealed a low-density area in the liver and dynamic contrast-enhanced MRI (EOB-MRI) showed that the tumor was completely washed out in the delayed phase. ^18^Fluorine-fluorodeoxyglucose positron emission tomography (FDG-PET) showed strong uptake in the liver. A diagnosis of liver metastasis was made and partial hepatectomy was performed. Microscopic examination showed that the tumor was an IPT.

**Conclusion:**

Differential diagnosis between IPT and malignant neoplasms is difficult. Moreover, FDG-PET revealed strong uptake in the tumor. To our knowledge, this is the first patient reported to have an IPT in association with a rectal GIST. This patient is discussed along with a review of the literature.

## Background

Inflammatory pseudotumor (IPT) is a rare benign tumor that may develop in various organs [1]. However, IPT of the liver is very rare and is often accompanied by fever, malaise, and abdominal pain. Due to the lack of characteristic clinical and radiological features for diagnosis, IPT may be mistaken as a malignant tumor, such as cholangiocarcinoma or metastatic cancer [2,3]. Thus, when computed tomography (CT) reveals a tumor of the liver in association with malignant disease, IPT of the liver is usually diagnosed as a metastatic tumor.

The medical history of the present case revealed that he had previously undergone a sacroabdominal rectal amputation for malignant GIST of the rectum. Moreover, ^18^fluorine-fluorodeoxyglucose positron emission tomography (FDG-PET) showed strong uptake in the liver, so a diagnosis was made of metastatic liver tumor of malignant GIST. IPT of the liver in association with GIST is very rare, with only two cases, including the present case, having been reported. Herein, we present our patient along with a discussion of the literature.

## Case presentation

A 74-year-old man was admitted to our hospital for examination and surgical treatment of a liver tumor. His medical history revealed that he had undergone a sacroabdominal rectal amputation for malignant GIST of the rectum at age 65 years. The resected specimen showed a tumor with intraluminal growth measuring 2 cm × 2.5 cm, with a center spot ulcer. The UICC TMN classification of GIST was T2N0M0. Upon histological examination, morphologic analysis suggested a spindle-cell GIST, and the diagnosis was confirmed by immunohistochemical (IHC) investigation. MIB-1 was strongly positive, but the surgical margin was negative. The final diagnosis was high-grade rectal GIST (mitotic index > 5/50 high power field). After surgery, the patient did not want to receive adjuvant chemotherapy and he underwent regular follow-up in our hospital according to National Comprehensive Cancer Network (NCCN) guidelines. The patient did not receive any other adjuvant therapy. The patient had never been outside of Japan.

**Figure 1:**
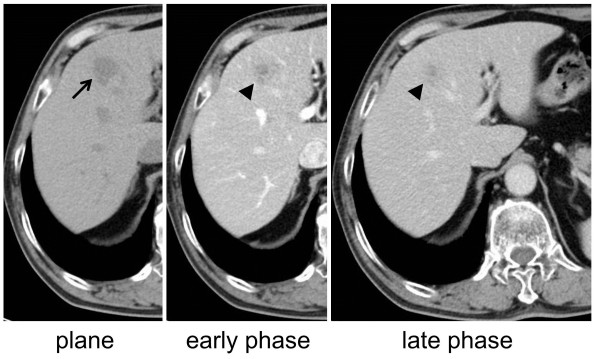
**Computed tomography (CT) findings nine years after the first surgery.** A 14-mm low-density area (arrow) in segment 8 of the liver (plane) is observed. The periphery of this area was nonhomogeneously enhanced by contrast medium and appeared to be isodense in the late phase (arrow head) compared to surrounding normal liver tissue.

**Figure 2:**
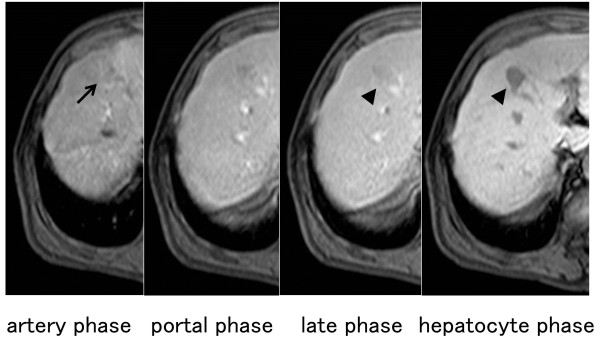
**Dynamic contrast-enhanced magnetic resonance imaging (MRI) findings with gadolinium ethoxybenzyl diethylenetriamine pentaacetic acid (EOB-MRI).** EOB-MRI showed that the tumor appears isointense in the arterial phase (arrow) and was completely washed out in the delayed and hepatocyte phases (arrow head).

**Figure 3:**
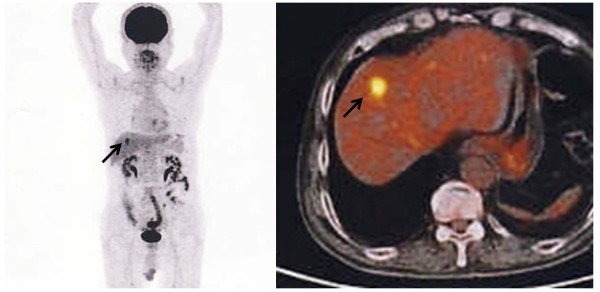
^**18**^**Fluorine-fluorodeoxyglucose positron emission tomography (FDG-PET) findings.** FDG-PET showed strong uptake in the liver (arrow). No other abnormal uptake was observed.

**Figure 4:**
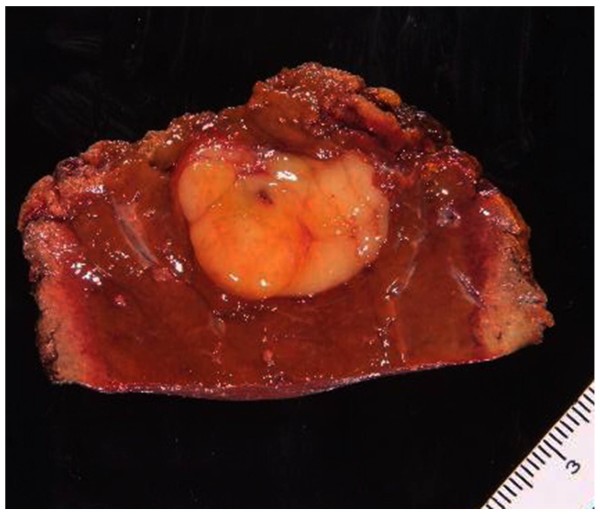
**A cross section of the resected liver.** The tumor was well-circumscribed, solid, and yellowish-white in color. No evidence of necrosis or hemorrhage was present.

**Figure 5:**
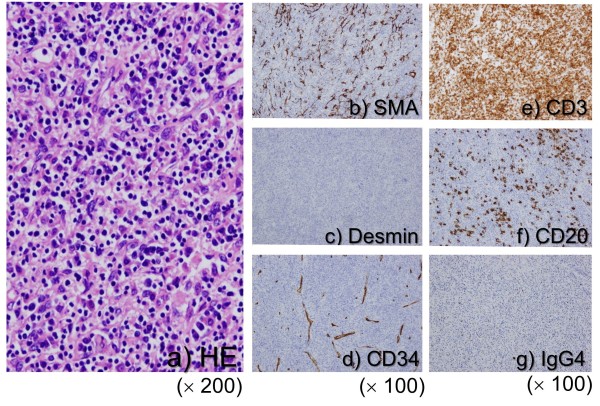
**Pathological findings. (a)** microscopic examination of the paraffin section of the liver ‘tumor’ showed that it was composed of fascicles of spindle cells such as fibroblasts and myofibroblasts accompanied by many lymphocytes, plasma cell, neutrophils, and macrophages. No mitotic cells were observed in these spindle cells (H&E stain, ×200). Immunohistochemical (IHC) studies of spindle cells showed positive staining for smooth muscle actin (SMA) **(b)** and negative staining for desmin **(c)** and CD34 **(d)**. IHC studies of white blood cells revealed that the majority of lymphocytes expressed CD3 **(e)** and some cells expressed CD20 **(f)**. Moreover, IHC showed some IgG4-positive plasma cells **(g)**.

Nine years after surgery, follow-up CT revealed a 14-mm low-density area in segment 8 of the liver. The periphery of this area was nonhomogeneously enhanced by contrast medium and appeared isodense in the late phase compared to surrounding normal liver tissue (Figure 1). Retrospectively, the tumor was just about identifiable on a CT scan acquired six months previously, and had grown bigger in the interim. Laboratory data were all within normal ranges, except for a slightly elevated C-reactive protein (CRP) level of 0.6 mg/dL. Hepatitis B surface antigen, hepatitis B e-antigen, and hepatitis C virus antibody were negative. Serum levels of carcinoembryonic antigen (CEA), carbohydrate antigen 19-9 (CA 19-9), and α-fetoprotein (AFP) were all within normal ranges. On dynamic contrast-enhanced magnetic resonance imaging (MRI) with gadolinium ethoxybenzyl diethylenetriamine pentaacetic acid (EOB-MRI), the tumor appeared to be isointense in the arterial phase and was completely washed out in the delayed and hepatocyte phases (Figure 2). There was no finding on both gastroscopy and colonoscopy. Based on these results, the tumor was suspected to be a metastatic tumor of the rectal GIST. FDG-PET was performed to confirm that the tumor was malignant, and showed strong uptake in the liver (Figure 3). No other abnormal uptake was observed. Surgical oncologists, medical oncologists, and radiologists discussed this case for multidisciplinary management. Considering the patient’s history combined with the radiological findings, a diagnosis of metastatic malignant GIST from the previous rectal lesion was made, and partial hepatectomy was performed. The tumor was well-circumscribed, solid, and yellowish-white in color (Figure 4). No evidence of necrosis or hemorrhage was present. Microscopic examination of the paraffin section of the liver ‘tumor’ showed that it was composed of fascicles of spindle cells, such as fibroblasts and myofibroblasts, accompanied by many lymphocytes, plasma cell, neutrophils, and macrophages (Figure 5). No mitotic cells were observed in these spindle cells. IHC studies of the spindle cells showed positive staining for smooth muscle actin (SMA) and vimentin, and negative staining for desmin, CD34, and cytokeratin AE1/3. Thus, the spindle cells were diagnosed as myofibroblasts. IHC studies of white blood cells revealed that the majority of lymphocytes expressed CD3 and some cells expressed CD20. Moreover, IHC revealed some IgG4-positive plasma cells. Since no evidence of malignancy was apparent, this case was diagnosed as IPT of the liver. The patient recovered well after surgery and was discharged without complications. He was followed-up regularly in our outpatient clinic, with half-yearly CT scans and blood tests. The patient remains asymptomatic and free of disease four years after his last surgery.

## Discussion

IPT of the liver is an uncommon, benign, tumor-like lesion that sometimes mimics a malignant tumor, particularly metastatic cancer or cholangiocarcinoma [4]. IPT of the liver, first described by Pack and Baker in 1953, is characterized by a well-circumscribed mass of chronic inflammatory cell infiltration and fibrosis [5]. It is very difficult to diagnose IPT of the liver due to the absence of specific symptoms, hematological abnormalities, and anomalous radiological findings. Patients with IPT sometimes present with fever, abdominal pain, abdominal discomfort, or leukocytosis, but these symptoms are not specific to IPT [6]. Radiological results for IPT are also inconsistent, because fatty depositions, tissue inflammation and necrosis, fibrosis, and bleeding can influence imaging [7]. Moreover, the degree and distribution of proliferating capillaries influence the staining patterns obtained by CT or MRI examination. Furthermore, IPT imaging findings are often similar to those of malignant tumors. In the present case, no symptoms or notable laboratory findings were present. The patient received regular follow-up, in accordance with his post-operative status of malignant rectal GIST. However, the liver lesion appeared suddenly. Therefore, liver metastasis from the rectal GIST was suspected. To confirm whether the liver lesion was malignant, FDG-PET was performed, which showed strong uptake in the liver with no other abnormal uptake observed; thus, hepatic resection was performed. In the past, patients with hepatic metastases from malignant GIST or leiomyosarcoma were considered to have a poor prognosis, even after surgical resection. However, researchers have recently advocated that aggressive surgical resection for liver metastasis might provide a survival benefit to these patients [8,9].

Rectal GIST is relatively rare compared to other types of GIST. Miettinen *et al*. reported the frequencies of GISTs as follows: stomach (60%), jejunum and ileum (30%), duodenum (4 to 5%), rectum (4%), and colon and appendix (1 to 2%) [10]. GIST is potentially malignant, depending on the tumor type (spindle versus epithelioid), tumor size, mitotic figures, necrosis, and Ki-67 labeling [11]. In the present case, histopathological findings revealed that the rectal GIST strongly expressed MIB-1 and was assigned a high grade. Thus, this tumor was highly suggestive of a GIST recurrence. Rectal GISTs metastasize predominantly to the liver, peritoneal surfaces, lungs, and bones, with the liver and peritoneal surfaces being most common [12]. Based on the high grade of the original tumor and the CT, MRI, and FDG-PET findings, this case was diagnosed as metastatic liver tumor. Nevertheless, after partial hepatectomy, the pathological findings revealed the tumor to be IPT of the liver.

Treatment for hepatic IPT remains controversial. Spontaneous regression occurs in some cases [13,14], and some IPT patients have also been successfully treated with conservative therapies such as corticosteroids [15] and antibiotics [16]. However, the differential diagnosis between malignant lesions, such as metastatic liver cancer and cholangiocarcinoma, and IPT is often difficult [17]. Thus, in the present case, surgical resection was considered to be indicated.

To our knowledge, this is the first patient reported to have an IPT associated with a rectal GIST. One previous report described a patient with IPT associated with a small bowel GIST [18]. In both cases, the ‘tumor’ was pre-diagnosed as a metastatic tumor from GIST, and hepatectomy was performed. The exact etiological link between the IPT and GIST is uncertain, although Lo *et al*. have hypothesized that the correlation may be related to inflammation [18]. The literature shows that in some cases, a percutaneous tumor biopsy provided the correct diagnosis. These patients were treated with antibiotics and/or corticosteroids, with complete resolution of the lesion; however, some of these lesions recurred [19]. In addition, Lo *et al*. suggested that biopsy was not sufficient to diagnose IPT of the liver, because the histological appearances of inflammatory pseudotumors and GISTs are particularly difficult to differentiate [18]. In contrast, numerous studies have reported hepatic resection to be employed, primarily due to the pre-operative malignant radiographic appearance of the tumor, and incidentally resected IPTs never recurred [20]. In the present case, in addition to the CT and MRI findings, FDG-PET showed strong uptake in the liver. These results suggest the possibility of malignancy. Recently, Park *et al*. summarized the experience of 45 IPT cases [21]. They insisted that to some extent, image finding was useful for a diagnosis. For example, enhanced CT scans indicated poorly defined peripheral enhancement at the arterial phase and poorly defined hyperattenuating lesions with internal hypoattenuating areas at the equilibrium phase. But these findings were not necessarily characteristic of IPT and did not accord with our case. Also, Park *et al*. insisted that histological diagnosis was necessary to make an accurate diagnosis of IPT. Percutaneous biopsy is useful for diagnosis, but possible coexistence of malignancy is sometimes not ruled out [21]. Furthermore, though IPTs are benign lesions, recurrences and malignant transformations were reported [22-25]. So Ntinas *et al*. concluded that hepatectomy is preferable because it not only minimizes the risk of a biopsy-related complication (dissemination in cases of malignancy), but it also abolishes the possibility of IPT recurrence [26]. However, there does appear to be a need for further research and discussion concerning the treatment of IPT of the liver.

## Conclusions

In conclusion, we experienced an extremely rare case of IPT of the liver associated with rectal GIST. In general, it is difficult to distinguish IPTs from malignant tumors by radiologic studies. Moreover, in the present case, FDG-PET showed strong uptake in the lesion. Since only two reports, including the present case, have described IPT in conjunction with GIST, further analysis of the etiology based on the accumulation of additional cases is required in the future. Surgeons should be aware of the possible association between these conditions, as illustrated in the present case.

## Consent

Written informed consent was obtained from the patient for the publication of this case presentation and any accompanying images. A copy of the written consent is available for review by the Editor-in-Chief of this journal.

## Abbreviations

IPT: Inflammatory pseudotumor; GIST: gastrointestinal stromal tumor; CT: computed tomography; MRI: magnetic resonance imaging; EOB-MRI: gadolinium ethoxybenzyl diethylenetriamine pentaacetic acid; FDG-PET: ^18^Fluorine-fluorodeoxyglucose positron emission tomography; IHC: Immunohistochemical; SMA: smooth muscle actin; NCCN: National Comprehensive Cancer Network; CRP: C-reactive protein; CEA: carcinoembryonic antigen; CA19-9: carbohydrate antigen 19-9; AFP: α-fetoprotein

## Competing interest

Yoichi Matsuo has no conflicts of interest to declare.

## Authors’ contributions

YM was responsible for the writing. MS, TS, and MM participated in data collection. KT, TS, and TH participated in literature searching. TS, HT and HT carried out the pathological examination. All authors have read and approved the final manuscript.
